# ICD-10 Diagnoses prior to ME/CFS diagnosis in children and young people suggest potential early diagnostic indicators

**DOI:** 10.1038/s41598-026-40848-1

**Published:** 2026-02-26

**Authors:** Marielle Wirth, Burkhard Haastert, Ute Linnenkamp, Silke Andrich, Andrea Icks, Rafael Pricoco, Uta Behrends, Freia De Bock

**Affiliations:** 1 Department of General Pediatrics, Neonatology and Pediatric Cardiology, Medical Faculty and University Hospital Düsseldorf, Heinrich Heine University Düsseldorf, Düsseldorf, Germany; 2 German Diabetes Center (DDZ), Institute for Biometrics and Epidemiology, Leibniz Center for Diabetes Research at Heinrich Heine University Düsseldorf, Düsseldorf, Germany; 3 mediStatistica, Wuppertal, Germany; 4 Centre for Health and Society, Institute for Health Services Research and Health Economics, Medical Faculty and University Hospital Düsseldorf, Heinrich Heine University Düsseldorf, Düsseldorf, Germany; 5 German Diabetes Center, Institute for Health Services Research and Health Economics, Leibniz Center for Diabetes Research at Heinrich Heine University Düsseldorf, Düsseldorf, Germany; 6 German Center for Diabetes Research, Partner Düsseldorf, München-Neuherberg, Germany; 7 Munich Chronic Fatigue Center for Young People (MCFC), TUM School of Medicine and Health, Children’s Hospital, Technical University of Munich, Munich, Germany; 8 Charité–Universitätsmedizin Berlin, corporate member of Freie Universität Berlin and Humboldt-Universität Zu Berlin, Institute of Medical Sociology and Rehabilitation Science, Berlin, Germany

**Keywords:** Myalgic encephalomyelitis, Chronic fatigue syndrome, ME/CFS, ICD-10-GM, Children and young people, Case–control study, Diseases, Health care, Medical research, Risk factors

## Abstract

To identify ICD-10-GM codes recorded in the year preceding a Myalgic Encephalomyelitis/Chronic Fatigue Syndrome (ME/CFS) diagnosis, we conducted a 1:5 matched case–control study using statutory health insurance data of 6–27-year-olds with ME/CFS (ICD-10-GM: G93.3, 2020–2022). Cases (n = 6,077) were matched 1:5 to controls by birth year, sex, and postal code. ICD-10-GM codes from the preceding year were analyzed using multivariable conditional logistic regression, reporting odds ratios (OR) and 95% confidence intervals. Most cases were female and aged 18–27 years. Forty-four ICD-10-GM code classes were associated with increased and four with decreased odds, spanning 13 diagnostic chapters. Most associations were in chapters F (mental/behavioral disorders), R (respiratory diseases), and M (musculoskeletal disorders). Frequent conditions included fatigue, depression, pain disorders, and somatoform disorders (≥ 10% in cases; ORs 1.11–2.19. Rare diagnoses (≤ 1% prevalence), such as fibromyalgia (OR 2.08, 95% CI: 1.20–3.59) and mild cognitive impairment (2.93, 1.21–7.10), were strongly associated. Four COVID-19 or vaccination-related code classes were identified, with post-COVID-19 condition showing the highest OR (3.84, 2.97–4.98). Several ICD-10-GM codes, including COVID-19 related codes, were associated with later ME/CFS diagnoses. Prospective studies should clarify timing relative to ME/CFS onset, and distinguish between pre-existing conditions, comorbidities, early manifestations, or misdiagnoses.

**Supplementary Information:**

The online version contains supplementary material available at 10.1038/s41598-026-40848-1.

## Introduction

Myalgic encephalomyelitis/chronic fatigue syndrome (ME/CFS) is a multi-system disease, characterized by dysfunctions in the immune, central nervous, and vascular systems, as well as alterations in cellular energy metabolism. Clinically, ME/CFS manifests with multiple symptoms, including chronic fatigue, post-exertional malaise (PEM), unrefreshing sleep, cognitive impairments, and/or orthostatic intolerance^1^. Due to the absence of universal biomarkers, ME/CFS diagnosis is based on clinical criteria such as the Canadian Consensus Criteria^2^, or the criteria of the former Institute of Medicine^3^. Thus, diagnosis requires a comprehensive clinical evaluation of key symptoms, along with physical examination, psychosocial evaluation, laboratory and functional investigations to rule out differential diagnoses with similar symptoms^1^.

This extensive and time-consuming diagnostic process reflects the complexity of the disease, which impairs all aspects of daily life for both affected individuals and their families^4^. Limited awareness, essential knowledge gaps, and especially a high symptom overlap between ME/CFS, functional somatic syndromes or fibromyalgia, can impede a timely and accurate diagnosis^5–8^.

In Germany, ME/CFS diagnosis are documented in routine healthcare records using the International Classification of Diseases 10^th^ Revision German Version (ICD-10-GM) code G93.3. In 2020, 0.09% of children and young people (CYP) statutorily insured by the Techniker Krankenkasse (TK) aged 6–27 years were newly diagnosed, increasing by 22.2 percent to 0.11% in 2022 (see Supplementary Table S1). This rise in diagnoses possibly reflects growing awareness of ME/CFS, particularly in the wake of the COVID-19 pandemic and its association with post-viral fatigue^9,10^.

Despite growing recognition of ME/CFS and its association with post-viral fatigue, the diagnostic process remains challenging. Little is known about potential early diagnostic indicators that may precede an official ME/CFS diagnosis. Identifying such indicators could help reduce delays in diagnosis, inform healthcare providers and contribute to an extended case definition approach in analyses of statutory health insurance data (SHI).

Given these challenges, we hypothesized that distinct ICD-10-GM codes in form of symptoms, clinical conditions, and patterns of healthcare utilization could be identified in SHI data that might be associated with later ME/CFS diagnosis. These early indicators may provide valuable insights into healthcare patterns and awareness, potentially facilitating earlier recognition and diagnosis.

## Methods

### Aim and study design

Our aim of this retrospective matched case–control study was to identify symptoms, clinical conditions and patterns of healthcare utilization in form of ICD-10-GM codes from the year preceding an ME/CFS diagnoses (G93.3) that could serve as early diagnostic indicators of ME/CFS for clinicians and health insurances. The reporting in this article follows the STROBE guideline for case–control studies^11^. As we used routinely collected statutory health insurance data, the Ethics committee deemed written informed consent. The study was conducted in accordance with the Declaration of Helsinki.

### Data source

We used nationwide SHI data from the Techniker Krankenkasse (TK), a large statutory health insurance fund in Germany, covering about 15% of the German population^12^. These data are routinely collected for reimbursement purposes and include information on demographics, inpatient and outpatient diagnoses and healthcare utilization.

Diagnoses are coded using ICD-10-GM and assigned to calendar quarters rather than exact dates. As a result, the chronological order of diagnoses within a quarter cannot be determined. Outpatient diagnoses are further classified by clinicians as confirmed or suspected. Inpatient diagnoses are recorded as main or secondary discharge diagnoses at the end of hospital stay and are considered confirmed diagnoses.

### Study population

We included CYP aged 6–27 years at the time of their index quarter, defined as the first calendar quarter in which an ME/CFS diagnosis was recorded (incident cases). Age was determined in the index quarter based on the year of birth. Eligible individuals were continuously insured at TK between January 1, 2019 and December 31, 2022, allowing for a maximum insurance gap of 30 days. We identified incident cases according to ICD-10-GM (G93.3), recorded between 2020 and 2022. These included outpatient diagnoses (confirmed, suspected) as well as inpatient main and secondary discharge diagnoses. Other health conditions or comorbidities were no exclusion criteria. We matched cases to controls in a 1:5 ratio, based on the year of birth, sex, and the first available postal code during the index quarter. We prioritized 5-digit postal codes; if unavailable, 3-digit postal codes were used. If fewer than five eligible controls were available for a given case, the maximum possible number of matches was selected. We assigned the same index quarter to the controls as their matched cases to ensure comparability in time periods and age. We randomly split the data into training and test subsets (80:20) for analyses.

### Variables

The exploratory variables analyzed included ICD-10-GM coded diagnoses from the year prior to the index quarter (hereafter referred to as exposure diagnoses) meeting the following criteria: (1) timing of diagnoses in the four quarters preceding the index quarter (not in the index quarter, as the order of diagnoses within a quarter is unclear); (2) outpatient as confirmed or suspected diagnosis; (3) inpatient as main and secondary discharge diagnoses at time of diagnoses between admission and discharge. After selection of all exposure diagnoses fulfilling criteria (1), (2), and (3) in the training data we excluded the ICD-10-GM coded diagnoses with exposure prevalence < 3% in the case group because these rare diagnoses in the cases would lead to a large number of covariates with imprecise estimations of associations in the models at the beginning of variable selection. We then grouped these into 119 ICD-10-GM code classes, each comprising either a single ICD-10-GM code or a combination of clinically related ICD-10-GM codes, based on clinical expertise (see Supplementary Table S2). For the presentation in this manuscript, we adapted the official classification. The dependent variable was case/control and the independent variables were the ICD-10-GM categories.

### Statistical analyses

We used descriptive statistics to characterize the study population, and estimated exposure prevalence and unadjusted odds ratios (OR) with 95% confidence intervals (CI) for the ICD-10-GM code classes, separately for cases and controls. We fitted multivariable conditional logistic regression models, using the matching sets as strata, and estimated ORs with 95% CIs as a measure of the differences in exposure prevalence between cases and controls. We investigated the 119 ICD-10-GM code classes of the primary model in the training dataset and selected variables with stepwise and backward procedures. Variables were included with a significance level of α = 0.10 and remained in the model with a significance level of α = 0.15^13^. By these variable selection procedures, we selected 65 variables. In addition, we performed explorative analyses using unconditional logistic regression with stepwise, backward, and Lasso selection and selected 10 additional ICD code classes. For this total of 75 ICD-10-GM code classes assessed, we compared models without relevant differences on the training and test data. Finally, we fitted the conditional logistic regression model with 75 ICD-10-GM code classes in the full dataset and the final model reduced to the 48 ICD-10-GM code classes with significant ORs (R^2^ = 0.096; max-rescaled-R^2^ = 0.1532). No subgroup or sensitivity analyses were conducted. We used Statistical Analysis Systems SAS 9.4 SAS/STAT 15.3 to perform the analyses (SAS Institute Inc. Cary, NC, USA).

## Results

### Study population

Out of 7,130,222 eligible individuals, 6,077 cases and 30,255 controls were included, with 43.0% having their index quarter in 2022, 31.4% in 2021 and 25.6% in 2020 (Table 1). They were predominantly female (65.7%) and primarily 18–24 years old (48.1%). The majority of the cases had five matched controls (98.5%).

**Table 1:** Sample characteristics of the ME/CFS cases and their controls, Germany, 2020–2022.

Characteristics	Cases	Controls
	*N (%)*	*N (%)*
*N*	6,077 (100.0%)	30,255 (100.0%)
*Index quarter*		
*2020*	1,558 (25.6%)	7,759 (25.7%)
*2021*	1,906 (31.4%)	9,489 (31.4%)
*2022*	2,613 (43.0%)	13,007 (43.0%)
*Sex*		
*Male*	2,084 (34.3%)	10,367 (34.3%)
*Female*	3,993 (65.7%)	19,888 (65.7%)
*Age [years]*		
*Mean (min; max)*	21.1 (6.0; 27.0)	21.1 (6.0; 27.0)
*6–12*	376 (6.2%)	1.,49 (6.1%)
*13–17*	987 (16.2%)	4,901 (16.2%)
*18–24*	2,923 (48.1%)	14,571 (48.2%)
*25–27*	1,791 (29.5%)	8,934 (29.5%)
*Matching pairs*	*N (%)*	
*5 controls*	5,985 (98.5%)	
*4 controls*	70 (1.2%)	
*3 controls*	11 (0.2%)	
*2 controls*	6 (0.1%)	
*1 control*	5 (0.1%)	

Deviations from 100% due to rounding.

### ICD categories and diagnostic classes associated with ME/CFS

**Fig. 1:**
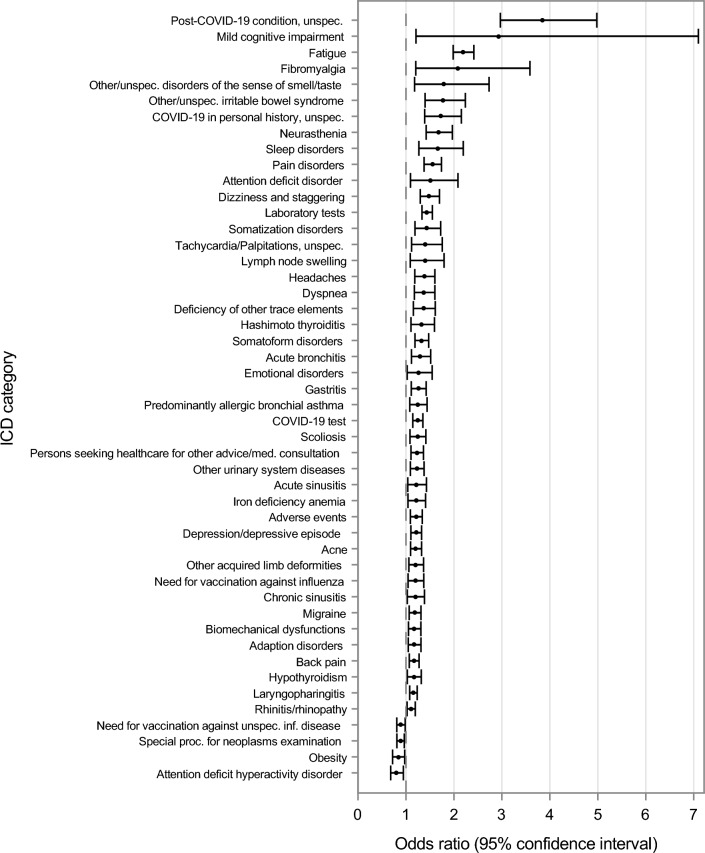
ICD-10-GM code classes associated with a later ME/CFS diagnosis in children and young people (6–27 years), Germany, 2020–2022.

The results of the univariate analyses of the 119 ICD-10-GM code classes are presented in Supplementary Table S2 and the results of the final model in Table 2 and displayed in the Forest plot in Fig. 1.

**Table 2:** ICD code classes associated with a first ME/CFS diagnosis in individuals aged 6–27 years.

ICD chapter	ICD code class	Description	Crude prevalence (95% CI)		Adjusted^a^ OR (95% CI)
Mental, behavioral and neurodevelopmental disorders	F32, F33	Depression/depressive episode	cases	16.26 (15.34–17.21)	1.21 (1.10–1.33)
			controls	9.45 (9.12–9.78)	
	F452, F453, F458, F459	Somatoform disorders	cases	11.77 (10.97–12.60)	1.32 (1.19–1.47)
			controls	5.41 (5.16–5.67)	
	F432	Adaption disorders	cases	9.74 (9.01–10.51)	1.17 (1.05–1.31)
			controls	5.51 (5.25–5.77)	
	F480	Neurasthenia	cases	5.30 (4.75–5.89)	1.67 (1.42–1.97)
			controls	1.72 (1.58–1.87)	
	F450, F451	Somatization disorders	cases	4.00 (3.52–4.52)	1.43 (1.19–1.72)
			controls	1.34 (1.22–1.48)	
	F900	Attention deficit hyperactivity disorder	cases	3.67 (3.21–4.17)	0.80 (0.68–0.95)
			controls	3.35 (3.15–3.56)	
	F930, F933, F938, F939	Emotional disorder	cases	2.67 (2.28–3.10)	1.26 (1.02–1.55)
			controls	1.61 (1.47–1.76)	
	F510, F512, F518, F519	Sleep disorders	cases	1.71 (1.40–2.07)	1.67 (1.27–2.19)
			controls	1.61 (1.47–1.76)	
	F9880	Attention deficit disorder	cases	1.15 (0.90–1.45)	1.51 (1.09–2.08)
			controls	0.52 (0.44–0.61)	
	F067	Mild cognitive impairment	cases	0.30 (0.18–0.47)	2.93 (1.21–7.10)
			controls	0.04 (0.02–0.07)	
Diseases of the respiratory system	J02, J06	Laryngopharingitis	cases	44.03 (42.78–45.29)	1.15 (1.08–1.23)
			controls	32.29 (31.76–32.82)	
	J00, J30	Rhinitis/rhinopathy	cases	22.79 (21.74–23.87)	1.11 (1.02–1.20)
			controls	15.83 (15.42–16.24)	
	J450	Predominantly allergic bronchial asthma	cases	5.55 (4.98–6.15)	1.25 (1.08–1.44)
			controls	3.15 (2.96–3.36)	
	J32	Chronic sinusitis	cases	5.12 (4.58–5.70)	1.19 (1.03–1.39)
			controls	2.78 (2.59–2.97)	
	J20	Acute bronchitis	cases	4.69 (4.17–5.25)	1.30 (1.11–1.51)
			controls	2.61 (2.43–2.80)	
	J01	Acute sinusitis	cases	4.46 (3.95–5.01)	1.22 (1.04–1.43)
			controls	2.40 (2.23–2.57)	
Symptoms, signs, and abnormal clinical and laboratory findings, not elsewhere classified	R53	Fatigue	cases	15.34 (14.44–16.27)	2.19 (1.98–2.42)
			controls	4.84 (4.60–5.09)	
	R42	Dizzines and staggering	cases	7.36 (6.71–8.04)	1.48 (1.30–1.70)
			controls	2.78 (2.59–2.97)	
	R060, R062, R068	Dyspnea	cases	5.84 (5.27–6.46)	1.37 (1.17–1.60)
			controls	2.04 (1.89–2.21)	
	R000	Tachycardia/Palpitations, unspecified	cases	2.70 (2.31–3.14)	1.40 (1.12–1.76)
			controls	0.94 (0.83–1.05)	
	R590, R591, R599	Lymph node swelling	cases	1.69 (1.39–2.05)	1.40 (1.09–1.80)
			controls	0.88 (0.78–0.99)	
	R438	Other and unspecified disorders of the sense of smell and taste	cases	0.77 (0.57–1.03)	1.79 (1.18–2.73)
			controls	0.21 (0.17–0.27)	
Diseases of the musculoskeletal system and connective tissue	M54	Back pain	cases	18.63 (17.66–19.63)	1.17 (1.07–1.27)
			controls	11.34 (10.99–11.71)	
	M99	Biomechanical dysfunctions, not elsewhere classified	cases	10.40 (9.64–11.19)	1.17 (1.05–1.31)
			controls	6.14 (5.88–6.42)	
	M21	Other acquired limb deformities	cases	6.50 (5.89–7.15)	1.20 (1.06–1.37)
			controls	4.39 (4.16–4.63)	
	M41	Scoliosis	cases	6.45 (5.85–7.10)	1.24 (1.08–1.42)
			controls	3.69 (3.48–3.90)	
	M797	Fibromyalgia	cases	0.56 (0.39–0.78)	2.08 (1.20–3.59)
			controls	0.10 (0.07–0.14)	
Factors influencing health status and contact with health services	Z017^1^	Laboratory tests	cases	72.08 (70.93–73.20)	1.44 (1.33–1.55)
			controls	56.93 (56.37- 57.49)	
	Z12	Special procedures for the examination of neoplasms	cases	20.80 (19.79–21.84)	0.88 (0.81–0.97)
			controls	19.84 (19.39–20.30)	
	Z269	Need for vaccination against unspecified infectious disease	cases	10.40 (9.64–11.19)	0.89 (0.81–0.98)
			controls	10.46 (10.12–10.81)	
	Z71	Persons seeking health care for other advice or medical consultation, not elsewhere classified	cases	10.24 (9.48–11.02)	1.23 (1.10–1.36)
			controls	6.76 (6.48–7.04)	
	Z251	Necessity of vaccination against influenza	cases	5.40 (4.84–6.00)	1.20 (1.04–1.37)
			controls	3.88 (3.67–4.10)	
Endocrine, nutritional, and metabolic diseases	E03	Hypothyroidism	cases	7.98 (7.31–8.69)	1.16 (1.03–1.32)
			controls	4.79 (4.55–5.04)	
	E66	Obesity	cases	4.21 (3.72–4.75)	0.84 (0.72–0.97)
			controls	3.77 (3.56–3.99)	
	E61	Deficiency of other trace elements	cases	4.15 (3.66–4.68)	1.36 (1.15–1.61)
			controls	1.97 (1.81–2.13)	
	E063	Hashimoto thyroiditis	cases	3.52 (3.07–4.02)	1.33 (1.11–1.59)
			controls	1.91 (1.76–2.07)	
Codes for special purposes	U071, U072, U073	COVID-19 test	cases	24.80 (23.72–25.90)	1.24 (1.14–1.35)
			controls	15.75 (15.34–16.17)	
	U09	Post-COVID-19 condition, unspecified	cases	3.67 (3.21–4.17)	3.84 (2.97–4.98)
			controls	0.41 (0.34–0.48)	
	U08	COVID-19 in personal history, unspecified	cases	3.42 (2.98–3.91)	1.73 (1.39–2.16)
			controls	0.93 (0.82–1.04)	
Diseases of the nervous system	G43	Migraine	cases	11.17 (10.39–11.99)	1.18 (1.07–1.31)
			controls	6.74 (6.46–7.03)	
	G44	Headaches	cases	5.53 (4.97–6.13)	1.38 (1.18–1.60)
			controls	2.36 (2.19–2.54)	
Diseases of the digestive system	K290, K291, K293, K294, K295, K296, K297, K298, K299	Gastritis	cases	7.47 (6.82–8.16)	1.26 (1.11–1.42)
			controls	4.06 (3.84–4.29)	
	K588	Other and unspecified irritable bowel syndrome	cases	2.24 (1.88–2.64)	1.77 (1.40–2.24)
			controls	0.80 (0.71–0.91)	
Diseases of the blood and blood-forming organs and certain disorders involving the immune mechanism	D50	Iron deficiency anemia	cases	4.77 (4.25–5.34)	1.21 (1.04–1.41)
			controls	2.71 (2.53–2.90)	
Diseases of the skin and subcutaneous tissue	L70	Acne	cases	11.95 (11.14–12.79)	1.21 (1.10–1.33)
			controls	8.99 (8.67–9.32)	
Diseases of the genitourinary system	N39	Other urinary system diseases	cases	8.08 (7.41–8.79)	1.23 (1.09–1.38)
			controls	5.16 (4.91–5.41)	
External causes of morbidity and mortality	T78	Adverse events, not elsewhere classified	cases	11.30 (10.52–12.13)	1.21 (1.09–1.34)
			controls	6.80 (6.51–7.08)	
Mixed chapter	F454, M790, M792, R072, R073, R074, R521, R522, R529	Pain disorders	cases	10.20 (9.45–10.99)	1.55 (1.38–1.74)
			controls	3.92 (3.70–4.14)	

Exposure prevalence and results of the multivariable conditional logistic regression analyses. ^a^Odds ratios are adjusted for each other ICD categories and (by matching) for age, sex, time period, and region. *N* = 6,077 cases, *N* = 30,255 controls insured by the Techniker Krankenkasse, 2019–2020, Germany. OR, odds ratio; CI, confidence interval.^1^Until 2020 ICD-Code UUU.

### Identified ICD-10-GM code classes and their distribution across diagnostic chapters

We identified 48 ICD-10-GM code classes (independent variables), including 44 ICD-10-GM code classes associated with increased odds, and four associated with decreased odds in cases compared to controls, respectively. These codes span 13 diagnostic chapters (e.g.; diseases of the respiratory system, assigned letter J), along with one additional pre-selected mixed chapter (pain disorders) (Table 2).

We identified the highest number of relevant ICD-10-GM code classes (n = 10) within the chapter F *(Mental and behavioral disorders)* and six code classes each in chapters J (*Diseases of the respiratory system),* R *(Symptoms, signs and abnormal clinical and laboratory findings, not elsewhere classified)* and M *(Diseases of the musculoskeletal system and connective tissue).* We found five categories in the chapter Z *(Factors influencing health status and contact with health services)* and four in chapter E *(Endocrine, nutritional and metabolic diseases).* We observed fewer code classes in the chapters U *(Codes for special purposes* (n = 3)) and G *(Diseases of the nervous system)* and K *(Diseases of the digestive system* (each n = 2)). We identified one code class in each of the following chapters: D *(Diseases of the blood and blood-forming organs and certain disorders involving the immune mechanism),* L *(Diseases of the skin and subcutaneous tissue),* N *(Diseases of the genitourinary system),* and T *(Injury, poisoning and certain other consequences of external causes).*

### ICD-10-GM code classes with a prevalence ≥ 10% among the cases

Ten ICD-10-GM code classes had a prevalence ≥ 10% among cases and were associated with a later documented ME/CFS diagnosis, with ORs ranging from 1.11 to 2.19. Nearly three-quarters of the cases underwent *laboratory tests* (Z017/UUU until 2020, OR 1.44, 95% CI 1.33–1.55), while almost half had *laryngopharingitis* (J02/J06, 1.15, 1.08–1.23), and nearly a quarter had *rhinitis/rhinopathy* (J00/J30, 1.11, 1.02–1.20). *Fatigue (R53)*, prevalent in 15.3% of the cases, was strongly associated with a later ME/CFS diagnosis (2.19, 1.98–2.42). Other code classes with a prevalence ranging from 10.0% to 12.0% included *depression/depressive symptoms* (F32/F33, 1.21, 1.10–1.33), *somatoform disorders* (F452/F453/F548/F459, 1.32, 1.19–1.47), *acne* (L70, 1.21, 1.10–1.33), *persons seeking health care for other advice or medical consultation, not elsewhere classified* (Z71, 1.24, 1.14–1.35), and *pain disorders* (M454/M790/M791/M792/R727/R374/R521/R522/R529, 1.55, 1.38–1.74).

### ICD-10-GM code classes with odds ratios between 1.5 and 2.0 comparing exposure prevalence of cases versus controls

Among the cases, three ICD-10-GM code classes were associated with approximately 1.7-fold increased odds of a later ME/CFS diagnosis compared to controls, with observed prevalence ranging from 1.7% to 5.3%: *neurasthenia* (F480, OR 1.67, 95% CI: 1.42–1.97), *sleep disorders* (F510/F511/F512/F518/F519, 1.67, 1.27–2.19), *other unspecified irritable bowel syndrome* (K588, 1.77, 1.40–2.24), and *other and unspecified disorders of the sense of smell and taste* (R438, 1.79, 1.18–2.73).

### ICD-10-GM code classes with odds ratios higher than 2.0 comparing exposure prevalence of cases versus controls

Two ICD-10-GM code classes were associated with more than a two-fold increase in the odds for a later ME/CFS diagnosis compared to controls (but with large CIs), with a prevalence below 1.0% in the cases: *mild cognitive impairment* (F067, OR 2.93, 95% CI 1.21–7.10) and *fibromyalgia* (M797, 2.08, 1.20–3.59).

### ICD-10-GM code classes with odds ratio below 1.0 comparing exposure prevalence in cases versus controls

Among the cases, an odds ratio of less than one was found for three ICD code classes, meaning that these ICD code classes were less likely to be documented for cases. *Special procedures for the examination of neoplasms* (Z12, 0.88, 0.81–0.97*)* was observed in 20.8% of the cases. *Obesity* (E66, 0.84, 0.72–0.97) and *attention deficit hyperactivity disorder* (F900, 0.80, 0.86–0.95) had prevalence ≤ 5.0%*.*

### ICD-10-GM code classes related to COVID-19 and vaccinations

We found four ICD-10-GM code classes related to COVID-19 testing and vaccinations, each showing varying prevalence and associations with later ME/CFS diagnosis. *COVID-19 testing (U071-U073)* had the highest prevalence at 24.8%, with a modest association (1.24, 1.14–1.35), followed by *necessity of vaccination against COVID-19, not elsewhere classified (U119)*, with a prevalence of 14.5% and a small but significant association (1.14, 1.07–1.27). The *need for vaccination against unspecified infectious diseases (U119)* was present in 10.4% of the cases, with a slightly negative association (0.89, 0.81–0.98). *Post-COVID-19 condition* (U09.9!) showed a strong association (3.84, 2.97–4.98), while *COVID-19 in personal history, unspecified* (U09.8) was moderately associated (1.73, 1.39–2.16) with a later ME/CFS diagnosis, with a prevalence of approximately 3.5% for both.

## Discussion

In this matched case–control study using SHI data, we identified a broad range of ICD-10-GM code classes that were associated with a later ME/CFS diagnosis. The majority of individuals had their first ME/CFS diagnosis coded in 2022, were female, and between 18–24 years old. Several pre-existing diagnoses – most notably from the domains of mental and behavioral disorders, respiratory diseases, and musculoskeletal conditions – were more common among cases than controls. Fatigue, pain disorders, and laboratory testing were among the most prevalent and strongly associated, and somatoform disorders and depression amongst the most prevalent diagnoses. Additionally, we found strong associations for less prevalent conditions such as fibromyalgia and mild cognitive impairment. While prior vaccinations against infections were negatively associated with a later ME/CFS diagnosis, we found a positive association with necessity of vaccination against COVID-19, preceding COVID-19 testing, and post-COVID-19 condition.

Based on these results, we were able to confirm our hypothesis, that the presence of distinct ICD-10-GM codes might be associated with later ME/CFS diagnosis and may thus help with early identification of patients at risk in SHI data. We identified a wide range of codes, many of which aligned with existing literature on early symptoms, comorbidities, or commonly reported features in diagnostic pathways. Up to 58.2% of cases, but also more than one third (35.4%) of controls had previously received diagnosis coded within at least four of the identified ICD-10-GM code classes.

As expected, documented fatigue, a hallmark of ME/CFS, was one of the findings strongly associated with a later diagnosis of ME/CFS^3,10^. Codes indicating preceding pain, another frequent symptom of ME/CFS or of fibromyalgia—a frequent comorbidity of ME/CFS – also showed a strong association^14,15^. In our dataset, irritable bowel syndrome, a functional somatic syndrome, was also recorded prior to the ME/CFS diagnosis. These records most likely reflect overlapping symptomatology^8^, and underscore that accurate and timely identification of ME/CFS is essential for guiding appropriate clinical management, rather than assigning multiple syndrome diagnoses to the same individual.

Evidence regarding the impact of psychiatric and psychosomatic conditions in the context of ME/CFS remains conflicting, and is subject to ongoing scientific and medical debate. Harvey et al., for example, identified shared risk factors as well as a temporal dose–response relationship between psychiatric disorders and the subsequent development of ME/CFS, suggesting a potential causal association^16^. In contrast, Jason et al. observed that psychological symptoms did not predict the later onset of ME/CFS^17^. Psychological symptoms in form of ICD-10-GM codes, however, might only be coded if antidepressant treatment is given and may be influenced by stigma.

Our findings indicate differential associations for attention-deficit disorder without hyperactivity (ADS) and hyperactivity (ADHD) preceding an ME/CFS diagnosis, with ADHD showing a prevalence similar to controls and an OR below 1, while ADS was more prevalent among cases (OR ~ 1.5). These divergent results highlight an important research gap regarding the possible relationship between neurodevelopmental disorders and ME/CFS in children and young people. Longitudinal evidence suggests that early neurodivergent traits, such as ADHD, are associated with an increased risk of developing chronic disabling fatigue at age 18^18^. However, the divergent associations may simply reflect differences in diagnostic practices in Germany. Hyperactive symptoms are often more notable and lead to earlier evaluation, whereas inattentive symptoms (ADS) may overlap with early fatigue and cognitive difficulties in ME/CFS, resulting in higher recorded prevalence prior to diagnosis.

Respiratory diseases caused by viral infections are known triggers of ME/CFS, particularly COVID-19, and pathophysiological mechanisms between ME/CFS and post-acute sequalae of COVID-19 might overlap^9,19^. In a study of adults with a prior SARS-COV-2 infection, 4.5% of individuals met the diagnostic criteria for ME/CFS, compared to only 0.6% in a non-infected control group^9^. Furthermore, a substantial proportion of individuals continue to experience symptoms such as chronic fatigue, cognitive impairment, headache, or dyspnea up to 12 months after COVID-19^20,21^.

Importantly, we found that prior vaccinations against infections were negatively associated with later ME/CFS diagnosis. This finding supports the evidence, that vaccinations might rather protect against ME/CFS than present relevant risk factors. For example, the risk for ME/CFS was not increased following influenza A vaccination, but following an influenza A infection^22^. Similarly, vaccination against polio showed no negative effects, not in individuals with ME/CFS or in individuals without ME/CFS^23^, and the risk for ME/CFS was not increased after a vaccination against human papilloma virus^24^. The risk long-term sequelae following COVID-19 vaccinations is not yet well defined^25^.

In summary, although SHI data might not reliably identify all ME/CFS cases^26,27^, several ICD-10-GM codes were identified in this study that could aid in the earlier recognition of individuals at risk. Such insights may help reduce unnecessary diagnostic procedures, facilitate timely diagnosis, and improve care coordination. Our findings thus contribute to the growing body of research on early ME/CFS recognition, and may support public health efforts to improve diagnostic pathways for ME/CFS.

## Strengths and limitations

To our knowledge, this is the first study investigating which ICD-10-GM-coded conditions precede a later ME/CFS diagnosis and occur with significantly higher prevalence in cases than in controls. A key strength of this study is the use of a large, nationwide dataset derived from routine clinical care, which made it possible to examine diagnostic patterns under real-world conditions. This comprehensive data source allowed for the identification of both common and rare pre-existing conditions as well as robust, positive and negative associations. Moreover, the matched case–control design with a large control group matched for regional and demographic factors, further strengthened internal validity by ensuring comparability between cases and controls.

Several limitations should be considered when interpreting these results. As our analysis was based on data from a single German SHI provider, the generalizability of the results may be limited. Further, we examined ICD-coded diagnoses documented within the 12 months preceding the first recorded ME/CFS diagnosis, based on quarterly data; precise timing of ME/CFS diagnosis within this period was unavailable. The marked increase in ME/CFS diagnoses observed in 2022 may reflect heightened awareness and/or coding practices during the COVID-19 pandemic, factors which cannot be further explored with the present dataset. In addition, routine healthcare utilization was likely reduced during 2020 and early 2021 due to pandemic-related restrictions and patients’ behavior. As a result, some diagnoses or healthcare encounters recorded in the year preceding an ME/CFS diagnosis may be underrepresented for this period. Furthermore, as the data were primarily generated for billing purposes, diagnostic coding practices may have been influenced by physicians’ administrative practices. Consequently, it cannot be ruled out that certain information, such as specific diagnoses were not captured in the dataset. Unmeasured confounding was also possible, as data on clinical severity, psychosocial factors, and health-seeking behavior was not available. Moreover, variable selection in regression models followed a data-driven approach and may have introduced bias; however, we sought to mitigate this by using separate training and test datasets. Finally, given the retrospective observational, and non-randomized design, the findings should be interpreted as associations rather than causal relationships. Prospective studies are needed to evaluate the predictive value of ICD codes, which may support the identification of undiagnosed ME/CFS cases in SHI data.

## Conclusion

Several pre-existing conditions, including mental and behavioral disorders, respiratory diseases, and musculoskeletal conditions were more prevalent among CYP later diagnosed with ME/CFS. Fatigue, pain disorder, depression, fibromyalgia, and COVID-19 related ICD codes were among the conditions most strongly associated with later ME/CFS diagnosis. Future studies should prospectively investigate the temporal relationship of these conditions to ME/CFS onset, and determine whether they represent pre-existing conditions, comorbidities, early manifestations, or misdiagnoses. Enhanced awareness and improved diagnostic practices are essential for the timely recognition and optional management of ME/CFS in CYP.

## Supplementary Information

Supplementary Information.

## Data Availability

The data that support the findings of this study are available from the Techniker Krankenkasse, a German health insurance company, but restrictions apply to the availability of these data, which were used under license for the current study, and so are not publicly available. Data are however available from the authors upon reasonable request and with permission of the Techniker Krankenkasse.

## Abbreviations

CI: Confidence interval
CYP: Children and young people
ICD-10-GM: International classification of diseases and related health problems, 10^th^ revision, German version
ME/CFS: Myalgic encephalomyelitis/chronic fatigue syndrome
OR: Odds ratio
SHI: Statutory health insurance
TK: Techniker krankenkasse
