# Supplementary material for: ICD-10 Diagnoses prior to ME/CFS diagnosis in children and young people suggest potential early diagnostic indicators
Source: Sci Rep. 2026 Feb 26;16:7736. doi: 10.1038/s41598-026-40848-1 (PMC12949141; doi:10.1038/s41598-026-40848-1)
Supplement: Supplementary file 1 — Supplementary Information. [file 41598_2026_40848_MOESM1_ESM.pdf]

Supplementary Table S1. Crude ME/CFS incidence rates per 10,000 person-years at risk, 2020-2022

	Number of first ME/CFS diagnoses	***Person-years at risk***	***ME/CFS diagnoses/ 10,000 pyrs (95% CI)***
2020	1,556	2,367,346	6.57 (6.25-6.90)
2021	1,901	2,371,582	8.02 (7.66-8.38)
2022	2,609	2,391,294	10.91 (10.49-11.33)

Data source: The same dataset as for the main analyses is used, namely the Techniker Krankenkasse, a large statutory health insurance fund covering approximately 15% of the German population. CI, confidence interval; ME/CFS, Myalgic encephalomyelitis/chronic fatigue syndrome.

Supplementary Table S2. ICD-10 code classes associated with a first ME/CFS diagnosis in individuals aged 6-27 years

ICD-10 class	ICD-10 category	Description	Crude Prevalence (95% CI)		Crude Odds ratio (95% CI)
Certain Infectious and Parasitic Diseases	A09, K52	Gastroenteritis	cases	16.11 (15.19-17.06)	1.411 (1.306- 1.525)
			controls	12.08 (11.71-12.45)	
	B34	Virus infection	cases	9.51 (8.79-10.28)	1.599 (1.448- 1.765)
			controls	6.22 (5.95-6.50)	
	B07	Viral warst	cases	3.44 (3.00-3.93)	1.188 (1.019- 1.386)
			controls	2.91 (2.72-3.10)	
	B37	Candidosis	cases	4.38 (3.88-4.92)	1.562 (1.354- 1.802)
			controls	2.89 (2.70-3.08)	
	B99	Other and unspecified infectious diseases	cases	4.94 (4.41-5.51)	1.481 (1.298- 1.690)
			controls	3.38 (3.18-3.59)	
Diseases of the Blood and Blood-forming Organs and Certain Disorders Involving the Immune Mechanism	D22	Melanocytic nevus	cases	8.79 (8.09-9.53)	1.286 (1.164- 1.421)
			controls	7.00 (6.72-7.29)	
	D50	Iron deficiency anaemia	cases	4.77 (4.25-5.34)	1.818 (1.583- 2.087)
			controls	2.71 (2.53-2.90)	
Endocrine, Nutritional, and Metabolic Diseases	E063	Hashimoto thyreoiditis	cases	3.52 (3.07-4.02)	1.897 (1.614- 2.228)
			controls	1.91 (1.76-2.07)	
	E559	Vitamin d deficiency, unspecified	cases	5.38 (4.83-5.98)	2.173 (1.902- 2.483)
			controls	2.56 (2.39-2.75)	
	E03	Hypothyreosis	cases	7.98 (7.31-8.69)	1.753 (1.573- 1.954)
			controls	4.79 (4.55-5.04)	
	E61	Deficiency of other trace elements	cases	4.15 (3.66-4.68)	2.174 (1.869- 2.529)
			controls	1.97 (1.81-2.13)	
	E73	Lactose intoleranz	cases	3.16 (2.73-3.63)	1.927 (1.628- 2.281)
			controls	1.66 (1.52-1.81)	
	E66	Obesity	cases	4.21 (3.72-4.75)	1.125 (0.979- 1.293)
			controls	3.77 (3.56-3.99)	
Mental, behavioral and neurodevelopmental disorders	F32, F33	Depression/depressive episode	cases	16.26 (15.34-17.21)	1.902 (1.756- 2.061)
			controls	9.45 (9.12-9.78)	
	F900	Attention deficit hyperactivity syndrome	cases	3.67 (3.21-4.17)	1.098 (0.946- 1.275)
			controls	3.35 (3.15-3.56)	
	F40_F931	Phobia	cases	3.75 (3.29-4.26)	1.548 (1.331- 1.800)
			controls	2.46 (2.29-2.64)	

	F930, F933, F938, F939	Emotional disorders	cases	2.67 (2.28-3.10)	1.732 (1.436- 2.089)
			controls	1.61 (1.47-1.76)	
	F989, F988, F988	Behavioral or emotional disorders with onset usually occurring in childhood and adolescence, unspecified	cases	1.40 (1.12-1.73)	1.689 (1.313- 2.171)
			controls	0.85 (0.75-0.96)	
	F42	Obsessive comulsive disorder	cases	1.71 (1.40-2.07)	1.940 (1.544- 2.439)
			controls	0.90 (0.79-1.01)	
	F9880	Attention deficit syndrome	cases	1.15 (0.90-1.45)	2.207 (1.659- 2.935)
			controls	0.52 (0.44-0.61)	
	F510, F511, F512, F518, F519	Sleep disorders	cases	1.71 (1.40-2.07)	3.133 (2.448- 4.011)
			controls	0.55 (0.47-0.64)	
	F450, F451	Somatisation disorders	cases	4.00 (3.52-4.52)	3.084 (2.623- 3.627)
			controls	1.34 (1.22-1.48)	
	F067	Mild cognitive impairment	cases	0.30 (0.18-0.47)	7.500 (3.613-15.570)
			controls	0.04 (0.02-0.07)	
	F430, F431, F438, F439	Post-traumatic stress disorders	cases	7.50 (6.85-8.19)	1.631 (1.461- 1.820)
			controls	4.76 (4.53-5.01)	
	F432	Adaption disorder	cases	9.74 (9.01-10.51)	1.870 (1.693- 2.065)
			controls	5.51 (5.25-5.77)	
	F41, F932	Anxiety disorder	cases	9.51 (8.79-10.28)	1.945 (1.759- 2.151)
			controls	5.18 (4.94-5.44)	
	F480	Neurasthenia	cases	5.30 (4.75-5.89)	3.232 (2.801- 3.729)
			controls	1.72 (1.58-1.87)	
	F452, F453, F458, F459	Somatoform disorders	cases	11.77 (10.97-12.60)	2.371 (2.159- 2.605)
			controls	5.41 (5.16-5.67)	
Diseases of the Nervous System	G43	Migraine	cases	11.17 (10.39-11.99)	1.771 (1.613- 1.944)
			controls	6.74 (6.46-7.03)	
	G44	Headache	cases	5.53 (4.97-6.13)	2.428 (2.125- 2.774)
			controls	2.36 (2.19-2.54)	
	G470, G471, G472, G478, G479	Sleep disorders	cases	4.53 (4.02-5.08)	2.380 (2.056- 2.754)
			controls	1.95 (1.79-2.11)	
Diseases of the Eye and Adnexa / Ear and Mastoid Process	H522	Astigmatism	cases	13.20 (12.36-14.07)	1.543 (1.417- 1.681)
			controls	9.09 (8.76-9.42)	
	H612	Ceruminous plug	cases	4.97 (4.44-5.55)	1.248 (1.096- 1.421)
			controls	4.04 (3.82-4.26)	
	H932	Other abnormal auditory sensations including hyperacusis	cases	0.59 (0.42-0.82)	1.933 (1.315- 2.843)
			controls	0.31 (0.25-0.38)	
	H521	Myopia	cases	8.70 (8.01-9.44)	1.402 (1.267- 1.551)
			controls	6.39 (6.11-6.67)	

	H10	Conjunctivits	cases	5.53 (4.97-6.13)	1.555 (1.372- 1.764)
			controls	3.63 (3.42-3.84)	
	H520	Hypermetrophy	cases	7.24 (6.60-7.92)	1.594 (1.422- 1.786)
			controls	4.79 (4.55-5.04)	
	H50	Strabismus	cases	5.20 (4.66-5.79)	1.510 (1.322- 1.723)
			controls	3.58 (3.37-3.80)	
Diseases of the Circulatory System	I951	Orthostatic hypotension/dysregulation	cases	1.45 (1.16-1.78)	2.046 (1.594- 2.628)
			controls	0.71 (0.62-0.82)	
Diseases of the Respiratory System	J02, J06	Laryngopharingitis	cases	44.03 (42.78-45.29)	1.684 (1.590- 1.784)
			controls	32.29 (31.76-32.82)	
	J00, J30	Rhinitis/Rhinopathy	cases	22.79 (21.74-23.87)	1.574 (1.471- 1.684)
			controls	15.83 (15.42-16.24)	
	J32	Chronic sinusitis	cases	5.12 (4.58-5.70)	1.917 (1.675- 2.194)
			controls	2.78 (2.59-2.97)	
	J450	Predominantly allergic bronchial asthma	cases	5.55 (4.98-6.15)	1.807 (1.590- 2.052)
			controls	3.15 (2.96-3.36)	
	J451, J458, J459	Exertion asthma	cases	9.36 (8.64-10.12)	1.746 (1.580- 1.928)
			controls	5.61 (5.35-5.87)	
	J03	Tonsillitis	cases	6.75 (6.13-7.41)	1.403 (1.252- 1.573)
			controls	4.94 (4.70-5.19)	
	J01	Acute sinusitis	cases	4.46 (3.95-5.01)	1.908 (1.654- 2.202)
			controls	2.40 (2.23-2.57)	
	J20	Acute bronchitis	cases	4.69 (4.17-5.25)	1.851 (1.609- 2.130)
			controls	2.61 (2.43-2.80)	
	J98	Other respiratory diseases	cases	3.93 (3.46-4.45)	1.560 (1.345- 1.809)
			controls	2.56 (2.39-2.75)	
Diseases of the Digestive System	K21	Gastro-oesophageal reflux disease	cases	3.19 (2.76-3.67)	2.159 (1.819- 2.563)
			controls	1.52 (1.39-1.66)	
	K29	Gastritis	cases	7.47 (6.82-8.16)	1.926 (1.721- 2.155)
			controls	4.06 (3.84-4.29)	
	K588	Other and unspecified irritable bowel syndrome	cases	2.24 (1.88-2.64)	2.884 (2.328- 3.573)
			controls	0.80 (0.71-0.91)	
	K59	Other functional intestinal disorders	cases	3.14 (2.72-3.61)	2.012 (1.696- 2.387)
			controls	1.60 (1.46-1.75)	
Diseases of the Skin and Subcutaneous Tissue	L70	Acne	cases	11.95 (11.14-12.79)	1.383 (1.267- 1.510)
			controls	8.99 (8.67-9.32)	
	L20, L30	Eczema	cases	12.36 (11.54-13.21)	1.284 (1.179- 1.399)
			controls	9.91 (9.57-10.25)	

Diseases of the Musculoskeletal System and Connective Tissue	M41	Skoliosis	cases	6.45 (5.85-7.10)	1.808 (1.605- 2.036)
			controls	3.69 (3.48-3.90)	
	M454, M790, M791, M792, R727, R374, R521, R522, R529	Pain disorders	cases	10.20 (9.45-10.99)	2.809 (2.536- 3.111)
			controls	3.92 (3.70-4.14)	
	M54	Back pain	cases	18.63 (17.66-19.63)	1.831 (1.698- 1.974)
			controls	11.34 (10.99-11.71)	
	M99	Biomechanical dysfunctions, not elsewhere classified	cases	10.40 (9.64-11.19)	1.789 (1.626- 1.969)
			controls	6.14 (5.88-6.42)	
	M255	Joint pain	cases	7.24 (6.60-7.92)	1.647 (1.473- 1.841)
			controls	4.54 (4.31-4.79)	
	M21	Other acquired limb deformities	cases	6.50 (5.89-7.15)	1.517 (1.351- 1.704)
			controls	4.39 (4.16-4.63)	
	M797	Fibromyalgia	cases	0.56 (0.39-0.78)	5.785 (3.523- 9.501)
			controls	0.10 (0.07-0.14)	
	M791	Myalgia	cases	2.81 (2.41-3.26)	2.165 (1.806- 2.595)
			controls	1.31 (1.18-1.44)	
	M53	Other diseases of the spine and back, not elsewhere classified	cases	4.23 (3.74-4.77)	1.928 (1.664- 2.233)
			controls	2.25 (2.09-2.43)	
	M796	Pain in the extremities	cases	4.67 (4.16-5.23)	1.806 (1.573- 2.074)
			controls	2.65 (2.47-2.83)	
	M629	Muscle disorders, unspecified	cases	2.29 (1.93-2.70)	1.660 (1.367- 2.016)
			controls	1.40 (1.27-1.54)	
	M77	Other enthesopathies	cases	2.91 (2.50-3.37)	1.509 (1.272- 1.790)
			controls	1.96 (1.81-2.12)	
	M62	Other muscular disorders	cases	4.77 (4.25-5.34)	1.845 (1.608- 2.117)
			controls	2.65 (2.47-2.84)	
Diseases of the Genitourinary System	N83	Non-inflammatory diseases of the ovary, the uterine tube and the lig. latum uteri	cases	3.04 (2.63-3.51)	1.423 (1.203- 1.683)
			controls	2.18 (2.02-2.36)	
	N86	Erosion and ectropion of the cervix uteri	cases	3.82 (3.35-4.33)	1.024 (0.883- 1.188)
			controls	3.74 (3.53-3.96)	
	N91	Menstruation that is absent, too light or too infrequent	cases	3.65 (3.20-4.16)	1.041 (0.897- 1.210)
			controls	3.52 (3.32-3.74)	
	N39	Other urinary system diseases	cases	8.08 (7.41-8.79)	1.644 (1.477- 1.830)
			controls	5.16 (4.91-5.41)	
	N89	Other non-inflammatory vaginal diseases	cases	25.00 (23.91-26.10)	1.126 (1.044- 1.214)
			controls	23.47 (22.99-23.95)	
	N92	Too heavy, to frequent or irregular menstruation	cases	11.16 (10.38-11.98)	1.308 (1.191- 1.437)

			controls	8.98 (8.66-9.30)	
	N94	Menstrual symptoms	cases	17.62 (16.67-18.61)	1.255 (1.158- 1.360)
			controls	15.05 (14.65-15.46)	
	N76	Other inflammatory diseases of the vagina and vulva	cases	7.92 (7.25-8.62)	1.366 (1.225- 1.524)
			controls	6.07 (5.80-6.34)	
	N30	Cystitis	cases	4.20 (3.71-4.73)	1.394 (1.208- 1.609)
			controls	3.07 (2.88-3.27)	
Congential malformations, deformations, and chromosomal abnormalities	Q66	Congential foot deformities	cases	9.54 (8.82-10.31)	1.476 (1.339- 1.627)
			controls	6.71 (6.43-6.99)	
Symptoms, Signs, and Abnormal Clinical and Laboratory Findings, Not Elsewhere Classified	R000	Tachycardia and unspecified palpitations	cases	2.70 (2.31-3.14)	2.952 (2.429- 3.587)
			controls	0.94 (0.83-1.05)	
	R104	Other and unspecified abdominal pain	cases	11.11 (10.33-11.92)	1.780 (1.622- 1.953)
			controls	6.60 (6.32-6.89)	
	R05	Cough	cases	5.53 (4.97-6.13)	1.690 (1.488- 1.919)
			controls	3.35 (3.15-3.56)	
	R11	Nausea and vomiting	cases	7.88 (7.22-8.59)	1.786 (1.602- 1.991)
			controls	4.62 (4.38-4.86)	
	R42	Dizziness and staggering	cases	7.36 (6.71-8.04)	2.798 (2.484- 3.152)
			controls	2.78 (2.59-2.97)	
	R50	Fever of other and unknown cause	cases	3.59 (3.13-4.09)	1.984 (1.690- 2.329)
			controls	1.86 (1.71-2.02)	
	R51	Headache	cases	15.71 (14.81-16.65)	1.850 (1.707- 2.004)
			controls	9.25 (8.93-9.58)	
	R53	Fatigue	cases	15.34 (14.44-16.27)	3.635 (3.325- 3.974)
			controls	4.84 (4.60-5.09)	
	R55	Unclear syncope	cases	1.71 (1.40-2.07)	1.950 (1.550- 2.452)
			controls	0.89 (0.79-1.00)	
	R060, R062, R068	Dyspnoa	cases	5.84 (5.27-6.46)	2.984 (2.609- 3.412)
			controls	2.04 (1.89-2.21)	
	R070	Sore throat	cases	4.00 (3.52-4.52)	1.522 (1.315- 1.761)
			controls	2.66 (2.49-2.85)	
	R071	Chest pain	cases	0.26 (0.15-0.43)	4.000 (2.073- 7.719)
			controls	0.07 (0.04-0.10)	
	R101	Pain in the upper abdomen	cases	3.65 (3.20-4.16)	1.910 (1.631- 2.236)
			controls	1.96 (1.80-2.12)	
	R103	Pain with localisation in other parts of the lower abdomen	cases	9.61 (8.88-10.38)	1.478 (1.339- 1.633)
			controls	6.84 (6.56-7.13)	
	R231	Paleness	cases	0.12 (0.05-0.24)	1.826 (0.767- 4.343)

			controls	0.06 (0.04-0.10)	
	R430	Anosmia	cases	0.38 (0.24-0.57)	2.483 (1.505- 4.097)
			controls	0.15 (0.11-0.20)	
	R432	Parageusia	cases	0.20 (0.10-0.34)	2.590 (1.278- 5.249)
			controls	0.08 (0.05-0.11)	
	R438	Other unspecified disorders of the sense of smell and taste	cases	0.77 (0.57-1.03)	3.661 (2.507- 5.345)
			controls	0.21 (0.17-0.27)	
	R590, R591, R599	Lymph node swelling	cases	1.69 (1.39-2.05)	1.941 (1.543- 2.440)
			controls	0.88 (0.78-0.99)	
	R619	Hyperhidrosis, unspecified sweating	cases	0.92 (0.70-1.20)	2.197 (1.603- 3.011)
			controls	0.42 (0.35-0.50)	
	R634	Abnormal weight loss	cases	1.81 (1.49-2.18)	2.130 (1.700- 2.669)
			controls	0.86 (0.76-0.97)	
Injuries to Specific Body Regions	S93	Dislocation, sprain and strain of the joints and ligaments at the level of the upper ankle and foot	cases	3.55 (3.10-4.05)	1.101 (0.948- 1.280)
			controls	3.25 (3.05-3.45)	

External Causes of Morbidity and Mortality	T78	Undesirable side effects, not elsewhere classified	cases	11.30 (10.52-12.13)	1.757 (1.604- 1.926)
			controls	6.80 (6.51-7.08)	
	T88	Other complications of surgical procedures and medical treatment, not elsewhere classified	cases	5.36 (4.81-5.96)	1.674 (1.469- 1.907)
			controls	3.33 (3.13-3.54)	
	T14	Injury to an unspecified body region	cases	7.52 (6.87-8.21)	1.287 (1.157- 1.432)
			controls	5.95 (5.68-6.22)	
Codes for Special Purposes	U119	Necessity of vaccination against COVID-19, not elsewhere classified	cases	14.55 (13.67-15.46)	1.163 (1.066- 1.269)
			controls	13.06 (12.68-13.45)	
	U99	Special procedures for testing for SARS-CoV-2	cases	20.77 (19.75-21.81)	1.730 (1.605- 1.864)
			controls	13.83 (13.44-14.22)	
	U071, U072, U073	COVID 19 test	cases	24.80 (23.72-25.90)	1.883 (1.755- 2.020)
			controls	15.75 (15.34-16.17)	
	U09	Post-COVID-19 condition, unspecified	cases	3.67 (3.21-4.17)	9.583 (7.638-12.024)
			controls	0.41 (0.34-0.48)	
	U08	COVID-19 in personal history, unspecified	cases	3.42 (2.98-3.91)	3.916 (3.252- 4.715)
			controls	0.93 (0.82-1.04)	
Factors Influencing Health Status and Contact with Health Services	Z11	Special procedures for testing for infectious and parasitic diseases	cases	17.28 (16.34-18.25)	1.642 (1.518- 1.777)
			controls	11.63 (11.28-12.00)	
	Z71	Persons seeking health care for other advice or medical consultation, not elsewhere classified	cases	10.24 (9.48-11.02)	1.605 (1.457- 1.768)
			controls	6.76 (6.48-7.04)	
	Z241	Need for vaccination against viral encephalitis transmitted by arthropods	cases	3.59 (3.13-4.09)	1.056 (0.909- 1.227)
			controls	3.39 (3.19-3.60)	
	Z258	Need for vaccination against other specified individual viral diseases	cases	3.67 (3.21-4.17)	1.010 (0.868- 1.175)
			controls	3.62 (3.41-3.84)	
	Z014	General gynaecological examination (routine)	cases	10.65 (9.88-11.45)	1.149 (1.042- 1.267)
			controls	9.58 (9.25-9.92)	
	Z30	Contraceptive measures	cases	41.19 (39.95-42.44)	1.227 (1.133- 1.328)
			controls	38.70 (38.15-39.26)	
	Z017 (until 2020 UUU)	Laboratory tests	cases	72.08 (70.93-73.20)	2.254 (2.108- 2.409)
			controls	56.93 (56.37-57.49)	

	Z12	Special procedures fo the examination of neoplasms	cases	20.80 (19.79-21.84)	1.089 (1.004- 1.181)
			controls	19.84 (19.39-20.30)	
	Z269	Need for vaccination against unspecified infectious disease	cases	10.40 (9.64-11.19)	0.995 (0.908- 1.090)
			controls	10.46 (10.12-10.81)	
	Z20	Contact and exposure to communicable diseases	cases	5.10 (4.56-5.68)	1.733 (1.516- 1.982)
			controls	3.04 (2.85-3.24)	
	Z00	General examination and clarification for people without symptoms or a stated diagnosis	cases	12.06 (11.25-12.91)	1.415 (1.295- 1.547)
			controls	8.94 (8.62-9.27)	
	Z251	Necessity of vaccination against influenza, not elsewhere classified	cases	5.40 (4.84-6.00)	1.418 (1.250- 1.609)
			controls	3.88 (3.67-4.10)	

Results of the univariable analyses. n=6,077 cases, n=30,055 controls insured by the Techniker Krankenkasse, Germany, 2020-2022.
